# Role of mitochondria ROS generation in ethanol-induced NLRP3 inflammasome activation and cell death in astroglial cells

**DOI:** 10.3389/fncel.2014.00216

**Published:** 2014-08-01

**Authors:** Silvia Alfonso-Loeches, Juan R. Ureña-Peralta, Maria José Morillo-Bargues, Jorge Oliver-De La Cruz, Consuelo Guerri

**Affiliations:** Molecular and Cellular Pathology of Alcohol Laboratory, Prince Felipe Research CenterValencia, Spain

**Keywords:** ethanol, astrocytes, TLR4, NLRP3-inflammasome, ROS, pyroptosis, IL-β, apoptosis

## Abstract

Toll-like receptors (TLRs) and NOD-like receptors (NLRs) are innate immunity sensors that provide an early/effective response to pathogenic or injury conditions. We have reported that ethanol-induced TLR4 activation triggers signaling inflammatory responses in glial cells, causing neuroinflammation and brain damage. However, it is uncertain if ethanol is able to activate NLRs/inflammasome in astroglial cells, which is the mechanism of activation, and whether there is crosstalk between both immune sensors in glial cells. Here we show that chronic ethanol treatment increases the co-localization of caspase-1 with GFAP^+^ cells, and up-regulates IL-1β and IL-18 in the frontal medial cortex in WT, but not in TLR4 knockout mice. We further show that cultured cortical astrocytes expressed several inflammasomes (NLRP3, AIM2, NLRP1, and IPAF), although NLRP3 mRNA is the predominant form. Ethanol, as ATP and LPS treatments, up-regulates NLRP3 expression, and causes caspase-1 cleavage and the release of IL-1β and IL-18 in astrocytes supernatant. Ethanol-induced NLRP3/caspase-1 activation is mediated by mitochondrial (m) reactive oxygen species (ROS) generation because when using a specific mitochondria ROS scavenger, the mito-TEMPO (500 μM) or NLRP3 blocking peptide (4 μg/ml) or a specific caspase-1 inhibitor, Z-YVAD-FMK (10 μM), abrogates mROS release and reduces the up-regulation of IL-1β and IL-18 induced by ethanol or LPS or ATP. Confocal microscopy studies further confirm that ethanol, ATP or LPS promotes NLRP3/caspase-1 complex recruitment within the mitochondria to promote cell death by caspase-1-mediated pyroptosis, which accounts for ≈73% of total cell death (≈22%) and the remaining (≈25%) die by caspase-3-dependent apoptosis. Suppression of the TLR4 function abrogates most ethanol effects on NLRP3 activation and reduces cell death. These findings suggest that NLRP3 participates, in ethanol-induced neuroinflammation and highlight the NLRP3/TLR4 crosstalk in ethanol-induced brain injury.

## INTRODUCTION

Inflammation in the central nervous system (CNS), or neuroinflammation, is a key component of many neurological and neurodegenerative disorders characterized by lymphocyte/macrophage infiltration, glial activation, enhanced cytokine/chemokine production, demyelination and axonal loss (Sospedra and Martin, 2005; Pittock and Lucchinetti, 2007). The neuroinflammatory process is initiated by several receptors of the innate immune system, which recognize a diverse range of microbial and damage signals, coordinate protection and repair mechanisms.

Toll-like receptors (TLRs) and nucleotide-binding oligomerization domain (NOD)-like receptors (NLRs) are two major forms of innate immune sensors which provide immediate responses against pathogenic invasion, tissue injury and stress conditions. Both the TLRs and NLRs families of receptors are activated through the recognition of both conserved microbial structures, called pathogen-associated molecular patterns (PAMPs) and damage-associated molecular patterns (DAMPs). However, unlike membrane-bound TLRs that sense PAMPs or DAMPs on the cell surface or within endosomes, NLRs recognize microbial molecules or DAMPs in the host cytosol. Activation of these receptors induces the recruitment of innate immune cells, which initiates tissue repair processing and adaptive immune activation. Abnormalities in any of these innate sensor-mediated processes may cause excessive inflammation due to either hyper-responsive innate immune signaling or sustained compensatory adaptive immune activation.

NOD-like receptors family members, NLRP1, NLRP3, NLRC4, and AIM2 (a member of the PYHIN protein family), have been identified as being capable of forming inflammasomes, multiprotein complexes that activate caspase-1, which leads to the processing and secretion of pro-inflammatory cytokines interleukin-1β (IL-1β) and IL-18. Among NLRs, NLRP3 is currently the most fully described inflammasome. It consists of the NLRP3 scaffold, adaptor ASC (apoptosis-associated speck-like protein containing a CARD) and caspase-1. Nevertheless, the molecular and cellular mechanisms of NLRP3 activation remain unclear. Different mechanisms have been postulated, such as lysosomal damage (Hornung and Latz, 2010), potassium leakage (Arlehamn et al., 2010), and reactive oxygen species (ROS) formation (Schroder et al., 2010; Shimada et al., 2012a). Recent studies support the role of mitochondrial adaptors (Subramanian et al., 2013), mitochondria calcium fluxes (Triantafilou et al., 2013) and ROS formation (Zhou et al., 2011; Shimada et al., 2012b) in inflammasome activation.

NOD-like receptors P3 is activated upon exposure to whole pathogens, but also by a number of host-derived danger signals, which are indicative of not only tissue injury (DAMPs), but also environmental irritants (Schroder and Tschopp, 2010). Mutation of NLRP3 is responsible for rare autoinflammatory diseases, collectively referred to as cryopyrin-associated periodic syndromes (CAPS; Neven et al., 2004; Ting and Davis, 2005; Jha and Ting, 2009), characterized by the hyperactivation of the inflammasome complex and increased IL-1β (Neven et al., 2004; Goldbach-Mansky et al., 2006). In addition, there is emerging evidence for the participation of the NLRP3 inflammasome as a sensor of metabolic stress (i.e., De Nardo and Latz, 2011), demyelination, and it is also involved in some neurodegenerative disorders such as the multiple sclerosis model (Jha et al., 2010) and Alzheimer’s disease (Heneka et al., 2013; Sheedy et al., 2013; Tan et al., 2013).

Alcohol is a neurotoxic compound and its abuse can induce brain damage and neurodegeneration. We have demonstrated that ethanol is capable of activating TLR4/IL-1RI receptors in astroglial and microglial cells to trigger TLR4 signaling and to produce cytokines induction (IL-1β, TNF-α, IL-6) and inflammatory mediators (iNOS, COX-2), which can lead to neuroinflammation and brain injury. Elimination of the TLR4 function abolishes most neuroinflammation and neural death (Alfonso-Loeches et al., 2010). Our recent studies have further demonstrated that neuroinflammation induced by ethanol abuse in mice is associated with demyelination and disruptions in the myelin structure and that these alterations are, in part, dependent on TLR4 signaling (Alfonso-Loeches et al., 2012).

Crosstalk between TLRs and NLRs in the secretion of mature IL-1β during microbial infection has been reported (Kahlenberg et al., 2005; Becker and O’Neill, 2007; Mariathasan and Monack, 2007), although it is unknown whether this crosstalk occurs in brain damage and demyelination. A recent study shows that ethanol activates NLRP3 inflammasome in the brain (Lippai et al., 2013). However, the cellular and molecular mechanisms of ethanol-induced inflammasome activation in the brain and glial cells, and the interactions of TLRs and NLRs, are presently unknown. Here we present evidence for NLRP3 expression in astrocytes and that by stimulating mitochondria ROS (mROS) generation, ethanol, ATP or LPS triggers NLRP3/caspase-1 activation and the production of IL-1β and IL-18 in astrocytes. These findings further demonstrate that ethanol promotes NLRP3/caspase-1 co-localization within mitochondria to trigger pyroptosis and a small proportion of apoptosis. Elimination of the TLR4 function reduces the damaging actions of ethanol on NLRP3 inflammasome activation in astroglial cells in both primary culture and cerebral cortex *in vivo*, suggesting that TLR4 plays a key role in ethanol-induced NLRP3 activation, neuroinflammation and brain damage.

## MATERIAL AND METHODS

### MICE AND ETHANOL TREATMENT

Female C57BL/6 WT (Harlan Ibérica S.L., Barcelona) and TLR4 *knockout* (TLR4-KO) mice (C57BL/6 background, kindly provided by Dr. S. Akira, Osaka University, Japan) were used. Animals were kept under controlled light and dark conditions (12/12 h) at a temperature of 23°C and at 60% humidity. All the animal experiments were carried out in accordance with the guidelines set out in European Communities Council Directive (86/609/ECC) and Spanish Royal Decree 1201/2005. The experimental procedures were approved by the *Ethical Committee of Animal Experimentation of the Prince Felipe Research Center* (Protocol numbers 08-0060 and 08-0099) and were in accordance with the recommendations in the ARRIVE Guidelines for the care and use of experimental animals.

For chronic ethanol treatment, 40 (10 animals/group) 7-week-old C57BL/6 (WT/TLR4^+/+^) and TLR4-KO mice weighing 18–20 g were housed (four animals/cage), and were maintained with water (WT and TLR4-KO control) or water containing 10% (v/v) ethanol, and were placed on a solid diet *ad libitum* for 5 months.

### BRAIN TISSUE PREPARATION

Mice were deeply anesthetized by an intraperitoneal injection with sodium penthobarbital (60 mg/kg) and fentanyl (0.05 mg/kg) for analgesia. Animals were then transcardially perfused with 0.9% saline containing heparin (2 U/ml), immediately followed by 4% paraformaldehyde (PF) in 0.1 M phosphate buffer, pH 7.4, for tissue fixation to be post-fixed overnight at 4°C in 4% PF and stored in 30% sucrose solution at 4°C for cryoprotection. Coronal brain sections (40 μm) were obtained with a cryostat (Microm HM 505E) and were collected on polysine^TM^ glass slides (Menzel-Gläser, Thermo Scientific, Germany).

Some mice were killed by cervical dislocation; brains were removed, dissected using the mouse brain atlas coordinates (Franklin and Paxinos, 1997), and immediately snap-frozen in liquid nitrogen until used in the Western blot, RT-PCR, caspase-1 enzymatic activity and cytokines determination analyses.

### IMMUNOFLUORESCENCE

**FIGURE 1:**
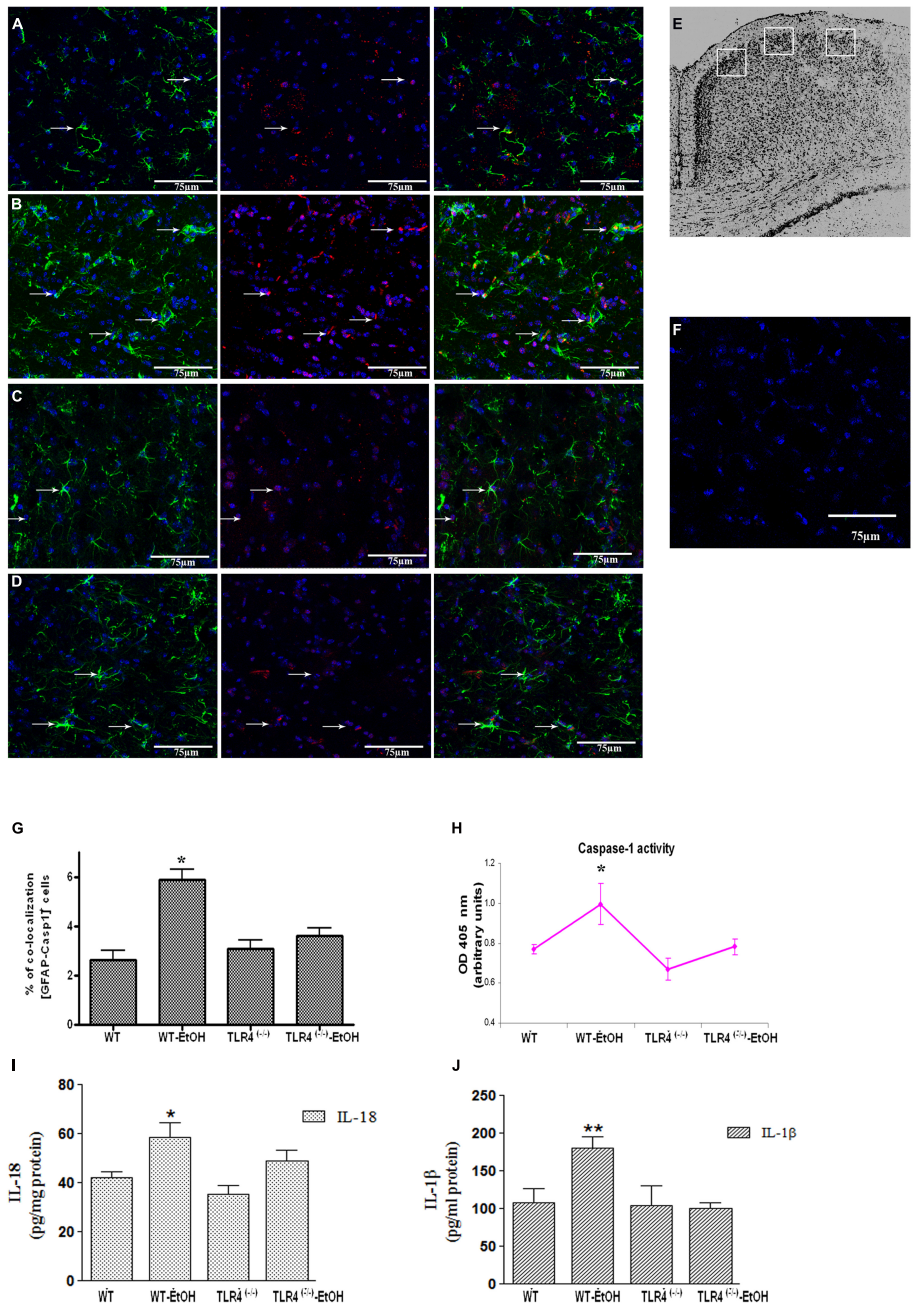
**Chronic ethanol intake increases caspase-1 activity in cortical astroglial cells of TLR4 mice.** **(A–D)** Confocal images illustrate the co-localization of caspase-1 (red) with GFAP (green) in the cortex area **(E)** of TLR4^+/+^ **(A,B)** and TLR4^-/-^ **(C,D)** mice (arrows indicate co-localization of the Casp-1 and GFAP-positive cells). **(F)** Confocal negative controls, in the absence of primary antibodies. We used four independent biological replicates from each experimental condition. Scale bar: 75 μm. **(G)** The quantitative analysis shows the percentage of the increased number of Casp-1/GFAP-positive co-localized cells in the cortices of ethanol-treated WT mice in relation to the untreated control mice. Non-significant changes were observed for the treated/untreated TLR4^-/-^ (TLR4-KO) mice. **(H)** Caspase-1 enzymatic activity was determined in the brain cortex. **(I,J)** The analysis of the IL-1β and IL-18 cytokines in the cortical homogenates of TLR4 mice was conducted by ELISA. Values represent the [mean ± SEM] of at least six to eight individual experiments.*p < 0.05, **p < 0.01 (Mann–Whitney *U* non-parametric test or a Student’s *t*-test).

(i) Double co-localization of caspase-1 and GFAP in cortical astrocytes*: cortical brain sections were defrosted for 30 min and incubated with 0.25% Triton in PBS solution for 10 min. Sections were blocked for 1 h/RT with 10% normal goat serum in TBS/T (0.1%) and were incubated overnight at 4°C with the following primary antibodies; anti-caspase-1 (1/50, Santa Cruz) and anti-GFAP (1/400, Sigma–Aldrich) following incubation with the respective Alexa-fluor-conjugated secondary antibodies (1/500, Invitrogen, Molecular Probes). Nuclei were stained with DAPI. Negative controls were performed by replacing the respective primary antibodies with IgG isotype control and concentration-matched irrelevant antibodies (**Figure 1F**). Sections were mounted onto glass slides with Dako Fluorescent Mounting medium (Dako North America Inc., CA, USA). All the images were analyzed with the ImageJ 1.42 software (NIH). We determined fluorescence intensity of three different animals per group by randomly taking four medial-frontal-cortex sections for each one. At least 800–1000 cells were counted per experimental condition. (ii) Double labeling of NLRP3-inflammasome and GFAP in cultured astrocytes*. Treated/untreated astroglial cells were previously incubated with 0.25% Triton in PBS solution for 10 min for cellular permeabilization. Cells were then blocked for 1 h at room temperature with 10% normal goat serum in TBS/T (0.1%), and were incubated overnight at 4°C with the following primary antibodies; anti-NLRP3 (1/150, AdipoGen) and anti-GFAP (1/400, Sigma–Aldrich), following incubation with the respective Alexa-fluor-conjugated secondary antibodies (1/500, Invitrogen, Molecular Probes). Nuclei were stained with DAPI. All the negative controls were also performed as described above *(i)*. Cells were mounted onto glass slides with Dako Fluorescent Mounting medium (Dako North America Inc., CA, USA), and confocal images were analyzed with the ImageJ 1.42 software (NIH). All the confocal images were acquired using the same settings, while fluorescence distribution was acquired with a Leica TCS-SP2-AOBA confocal laser-scanning microscope (Leica Microsystems, Heidelberg GmbH, Mannheim, Germany) using the 10× Plan-HCX PL APO CS10 and the 40× Plan-HCX PL APO CS40 × 1.25 oil objectives. All the confocal images were acquired with the same settings and the fluorescence distributions were analyzed by the Leica Confocal Software “Leica Lite,” version 2.61.

### CASPASE-1 ASSAY ACTIVITY

Caspase-1 activity was measured by the Colorimetric Assay Kit (Abcam). This assay allows the detection of chromophore *p*-nitroanilide (*p*NA) after cleavage from labeled substrate YVAD-*p*NA, performed following the manufacturer’s instructions. *p*NA light emission was quantified by a spectrophotometer at 405 nm. The comparison of the absorbance of *p*NA from treated vs. untreated controls allowed the determination of the fold increase in caspase-1 activity.

### PRIMARY CULTURE OF ASTROCYTES AND TREATMENTS

Primary cultured astrocytes of mice cortices (WT or TLR4-KO) from newborn pups were prepared and characterized as previously described (Minana et al., 2000). Cells were plated on 60-mm diameter plates in Dulbecco’s modified Eagle’s medium (DMEM, Gibco Invitrogen Corporation, Barcelona) containing 20% FBS, supplemented with L-glutamine (1%), glucose (1%), fungizone (1%), and antibiotics (1%). Cultures were grown in a humidified atmosphere of 5% CO_2_/95% air at 37°C. After 1 week of culture, FBS was reduced to 10%, and the medium was changed twice a week. Cells were grown to confluence and were used after 12 days in culture. The purity of astrocytes was assessed by immunofluorescence using: anti-glial fibrillary acidic protein (anti-GFAP, astrocyte marker, Sigma-Aldrich, Madrid, Spain), anti-CD11b (microglial marker, Serotec, Bionova, Madrid, Spain), anti-myelin basic protein (MBP, olygodendroglial marker, Sigma–Aldrich, Madrid, Spain) and anti-microtubule-associated protein 2 (MAP-2, neuronal marker, Sigma–Aldrich, Madrid, Spain). Astrocyte cultures were found to be at least 98% GFAP-positive and 2% CD11b-positive.

To assess the effect of ethanol (EtOH; 10 or 50 mM), lipopolysaccharide (LPS; 50 ng/ml) or ATP (5 mM), these compounds were added to DMEM in the absence of serum, but in the presence of 1 mg/ml bovine serum albumin. After 24 h of LPS, ATP or ethanol treatments, cells were harvested by trypsinization, centrifuged and used for specific determinations. The medium was used for cytokine determination.

In some experiments, astrocytes were incubated with a medium containing Z-YVAD-FMK (10 μM), a potent cell permeable and irreversible caspase-1 inhibitor (ICE; Parajuli et al., 2013; Tseng et al., 2013) or with Z-VAD-FMK (20 μM), a pan-caspase inhibitor (Dostert et al., 2009) or NLRP3 blocking peptide (bp; 4 μg/ml; Abcam) or Mito-TEMPO (500 μM), a ROS scavenger, for 30 or 60 min (for Mito-TEMPO) before and during ethanol (50 mM), or with ATP (5 mM) or LPS (50 ng/ml) treatments. Caspase-1 enzymatic activity, ROS release, IL-1β and IL-18 cytokines were determined in cells after 24 h treatments. Sterile toxin-free culture materials were used for all the experiments. In addition, the possible contamination of ethanol with LPS was determined by using the chromogenic limulus amebocyte lysate test, following the manufacturer’s instructions (Lonza Verviers SPRL). The endotoxin content in ethanol solution was 2.98 × 10^3^ pg/ml, which is far below the concentration required to induce astroglial activation under our assay conditions, as previously described (Alfonso-Loeches et al., 2010).

### FLUORESCENCE-ACTIVATED CELL SORTING (FACS)

#### MitoSOX^TM^ red mitochondrial superoxide indicator

To evaluate superoxide production by mitochondria, the MitoSOX^TM^ Red reagent (1.25 μM, Molecular Probes) was used in live cells, a fluorogenic dye specifically targeted to mitochondria in live cells. This reagent is readily oxidized by superoxide, but not by other ROS- or reactive nitrogen species (RNS)-generating systems. Oxidation of MitoSOX^TM^ Red reagent by superoxide produces red fluorescence. For these experiments, astrocytes were incubated for 24 h with different treatments; ethanol (10, 50 mM), LPS (50 ng/ml), ATP (5 mM) or rotenone (25 μM) as the positive controls, and with inhibitors Z-YVAD-FMK (Calbiochem) or NLRP3 bp or Mito-TEMPO. Then cells were further incubated with MitoSOX^TM^ (1.25 μM) for 30 min at 25°C in the darkness. Cell samples were acquired with a Cytomics FC500 flow cytometer (Beckman-Coulter, USA) equipped with 488 and 635 nm lasers detectors for forward scatter (FS) and side scatter (SS). We defined the cell population with the FS and SS parameters, MitoSOX Red was excited by laser at 488 nm, and its fluorescence emission was collected at 620 nm. In order to perform the analysis in live cells, a gate in the dot plot FS vs. SS was used, where cell debris was gated out for the analysis. We also used the LIVE/DEAD^®;^ Fixable Near-IR Dead Cell Stain Kit (Molecular Probes) to determine the viability of cells, and only the live cell population was used for the analysis. Data were represented as the [mean ± SEM], showing the percentage of gated mean intensity of MitoSOX fluorescence expressed as a percentage when comparing untreated or DMSO vehicle control-cells with MitoSOX positive-cells.

### AN IN CELL ANALYZER FOR CELLULAR DEATH DETECTION

The six-well plated cultured cells were incubated with the red caspase-1 detection probe, 660-YVAD-FMK (FLICA) following the manufacturer’s instructions (ImmunoChemistry Technologies, USA). Cells were washed with 4°C PBS before adding the 500 μl Annexin-binding buffer containing 2.5 μl of Annexin-V fluorescein isothiocyanate (FITC) and Hoechst (5 μg/ml) for 30 min of incubation. Finally, we added 1 μl propidium iodide (PI) solution for a further 5-min incubation in the dark. The stained samples were imaged live in an In Cell Analyzer 1000 high-content analysis system (GE Healthcare Life Sciences) equipped with a CCD camera and a 10×/0.45 NA objective. The 51008 polychroic mirror set was used in conjunction with the following excitation (×) and emission (m) filter combinations: 405/20×, 535/50m for Hoechst 33342, 475/20×, 535/50 m for Annexin-V FITC, 475/20×, 620/60 m for PI and 620/60×, 700/75 m for FLICA 660-YVAD-FMK. For the analysis, we defined a segmentation based on nuclei staining with Hoechst 33342. Then we established four groups in order to classify five populations: (1) Live cells were defined as negative cells for PI and normal staining with Hoechst 33342; (2) Apoptotic cells were defined as negative cells for PI and nuclei with increased staining with Hoechst 33342 (due to DNA condensation); (3) Necrotic cells were defined as positive for PI and negative for FLICA 660-YVAD-FMK staining; (4) pyroptotic cells were defined as double positive for PI and FLICA 660-YVAD-FMK. Twenty fields were acquired for each well. Four different experiments were performed and approximately 2000–10,000 cells were analyzed per experimental condition. The analysis was performed in the In Cell Analyzer 1000 Workstation software using the Multi Target Analysis Module.

### MITOTRACKER LIVE CELLS STAINING AND CO-LOCALIZATION STUDIES IN ASTROGLIAL CELLS

The astrocytes in primary culture were incubated for 10 min at 37°C with the MitoTracker^®^ Red CMXRos probe (50 nM, Molecular Probes, USA). This probe passively allows diffusion across the plasma membrane and is accumulated in active mitochondria. Cells were then treated with an aldehyde-based fixative, 3.7% of paraformaldehyde to allow further sample processing. For the co-localization studies, the cells stained with MitoTracker were immunostained with NLRP3 and with caspase-1 activity labeling. For this end, cells were incubated for 10 min in PBS containing 0.1% Triton X-100, rinsed off with PBS (1×) and blocked for 1 h/RT with 10% normal goat serum in TBS/T (0.1%). After blocking, sections were incubated with the mouse monoclonal anti-NLRP3 (1/150, AdipoGEN) followed by Alexa fluor 405 (1/500, Molecular Probes). Then sections were incubated with caspase-1 fluorescence activity using the FAM-FLICA^TM^ Caspase-1 assay kit (ImmunoChemistry Technologies, USA). For this labeling, cells were incubated for 1 h at RT with the fluorescent FAM-YVAD-FMK FLICA reagent following the manufacturer’s instructions (ImmunoChemistry Technologies, USA). The reagent becomes covalently coupled to the active enzyme and is retained within the cell, while the unbound FAM-YVAD-FMK FLICA reagent diffuses out of the cell and is washed away. The remaining green fluorescent signal is a direct measure of active caspase-1 enzyme activity. All the negative controls were performed by replacing the respective primary antibodies with isotype and concentration-matched irrelevant antibodies.

Sections were mounted onto glass slides with FluorSave Reagent (Calbiochem, USA). All the images were acquired using the same settings under a Leica TCS-SP2-AOBA confocal laser-scanning microscope (Leica Microsystems, Heidelberg GmbH, Mannheim, Germany) employing the 63× N.A. 1.4 oil objective. All the confocal images were acquired with the same settings and fluorescence distribution was analyzed by the Leica Confocal Software “Leica Lite,” version 2.61. Graphs represent the number of cellular co-localizations expressed as a percentage (%) of mitochondria co-localized with the NLRP3 receptor or the NLRP3 receptor with caspase-1 activity after the different treatments had been applied.

### CYTOKINES DETECTION

The supernatant medium from the 24 h-treated astrocytes and lysates from the brain cortices were used for cytokine determinations. Brain tissue was homogenized in cold lysis buffer (1% NP-40, 20 mM Tris-HCl, pH 8, 130 mM NaCl, 10 mM NaF, 10 μg/ml aprotinin, 10 mg/ml leupeptin, 10 μM DTT, 1 mM Na_3_VO_4_ and 1 mM PMSF; 250 mg tissue/0.5 ml). Lysis samples were kept on ice for 30 min and were centrifuged at maximum speed for 15 min, and the supernatant was collected for protein and cytokines determination. Protein was determined by the Bradford Assay (Bio-Rad, Hercules, CA, USA). The cytokine levels of IL-18 and IL-1β were measured using the enzyme-linked immunosorbent assay (ELISA) kits (Bender MedSystems GmbH, Austria) following the manufacturer’s instructions.

### WESTERN BLOT ANALYSIS

The protein extracts from the cortex and cultured astrocytes were homogenized in 250 mg tissue/0.5 ml cold lysis buffer (see above). Then they were kept on ice for 30 min, centrifuged at maximum speed for 15 min, and the supernatant was collected to determine the proteins levels using the Bradford Assay (Bio-Rad, Hercules, CA, USA). Lysates were separated by SDS-PAGE gels and were transferred to PVDF membranes following standard techniques. Membranes were blocked with 5% non-fat dried milk in TBS containing 0.1% Tween-20 (TBS-T). Next they were then incubated overnight with the following primary antibodies: anti-NLRP3 (1 μg/ml, Abcam), anti-caspase-1 (1/100, Santa Cruz Biotechnology); anti-pro-caspase-1 (1/200, Abcam); anti-Apaf-1 (1 μg/ml, Millipore Bioscience Research Reagents); anti-caspase-3 (1:500) and anti-caspase-9 (1:1000, Cell Signaling).

Some membranes were stripped for 1 h at 60°C in an SDS solution (2% SDS, 0.85% 2-ME, and 65 mM Tris-HCl, pH 6.8, and were washed and incubated with anti-GAPDH (1/3000, Chemicon) for 2 h as a loading control. The intensity of the bands was quantified with the image analysis program, α-Ease FC, version α Imager 2200 (Alpha Innotech Corporation).

### ASC OLIGOMERIZATION ASSAY

Apoptosis-associated speck-like protein containing a CARD pyroptosome were performed following the procedure of (Fernandes-Alnemri and Alnemri, 2008) with minor modifications. Thus, astrocytes were seeded in 50-mm diameter plates (1 × 10^6^ cells per well) and treated with different stimuli. Cells were pelleted by centrifugation and resuspended in 0.5 ml of ice-cold buffer containing PBS/Triton 0.5%, and lysed by shearing 10 times. Cell lysates were then centrifuged at 8000 × *g* for 15 min at 4°C, and the resultant pellets were washed twice with PBS and resuspended in 200 μl of PBS. The resuspended pellets, were then cross-linked with fresh disuccinimidyl suberate (DSS; 2 mM) for 30 min at room temperature, and pelleted by centrifugation at 8000 × *g* for 15 min. The cross-linked pellets were resuspended in 30 μl of SDS sample buffer, separated using 12% SDS-PAGE and immunoblotted employing anti-mouse ASC antibodies. The dimer band was quantified by densitometry.

### CO-IMMUNOPRECIPITATION OF NLRP3 AND CASPASE-1

Pre-cleared cell lysates were used for the co-immunoprecipitation (Co-IP) analysis using a Pierce Co-IP kit (Thermo Scientific Pierce) and following the manufacturer’s instructions. This kit provides covalent antibody immobilization onto an insoluble agarose support, and then incubated with a cell lysate containing the target protein. We use the purify mouse anti-NLRP3 antibody (10 μg, AdipoGEn) to immunoprecipitate the antigen (bait protein, IB) which bind the interacting protein (prey protein, IP) caspase-1 (1/100, Santa Cruz). Upon elution the immune complexes, the samples were resolved with 15% SDS-PAGE and transferred to PVDF membranes (Bio-Rad) for immunoblotting. The mouse IgG was used as a negative control.

### LACTATE DEHYDROGENASE (LDH) ACTIVITY

The release of LDH in the culture supernatant of astrocytes was measured using the colorimetric Cyto-Tox 96 Non-radioactive Assay (Promega) following the manufacturer’s instructions. This assay is a coupled enzymatic assay, which results in the conversion of a tetrazolium salt into a formazan product which, in turn, is proportional to the number of lysed cells. We used triton X-100 as a positive control. The LDH value was expressed as the percentage of total LDH activity, according to the following equation: %LDH release rate = 100 × [(Experimental - Effector Spontaneous - Target Spontaneous)/(Target Maximum - Target Spontaneous)].

### TERMINAL DEOXYNUCLEOTIDYL TRANSFERASE dUTP NICK END LABELING (TUNEL)

To assess cellular apoptosis (programmed cell death), we used the *in Situ* Cell Death Detection Kit (Roche), Fluorescein. In brief, coverslips with adherent cells were fixed and permeabilized (see Immunofluorescence) following incubation in a nucleotide mixture containing fluorescein-dUTP and TdT (terminal transferase), according to the manufacturer’s instructions. Cell nuclei were detected by incubation with DAPI. Micrographs were digitally recorded with a Leica DM6000 FS fluorescence microscope. TUNEL-positive cells appeared green, whereas TUNEL-negative nuclei appeared blue. Negative controls were incubated without TdT. The percentage of TUNEL-positive cells *vs*. TUNEL-negative cells was quantified for each experimental condition.

### RNA ISOLATION, REVERSE TRANSCRIPTION PCR AND QUANTIFICATION OF mRNA LEVELS

RNA was extracted from the cortex brain area using Trizol according to the manufacturer’s instructions (Sigma). RNA was measured in a NanoDrop ND-1000 Spectrophotometer (260/280 nm ratio). First-strand cDNA synthesis was performed with the *Transcriptor First Strand cDNA Synthesis Kit* (Roche Applied Biosystems) using 2000 ng of total RNA according to the manufacturer’s instructions. The RT-PCR reactions contained *LightCycler 480 SYBR Green I Master (2×*; Roche Applied Science), 5 μM forward and reverse primers, and 1 μl of cDNA. RT-PCR was performed in a LightCycler^®^ *480 System* (Roche). The amplification efficiency (*E*) of the primers was calculated from the plot of the Cq values against the cDNA input according to the equation *E* = [10(-1/slope)]. The relative expression ratio of a target/reference gene was calculated according to the Pfaﬄ equation (Pfaﬄ, 2001). The sequence of both the forward and reverse primers used in this study is detailed in **Table 1**. Housekeeping cyclophilin A (PPIA) was used as an internal control. Fluorescence was recorded in the annealing/elongation step in each cycle. A melting curve analysis was performed at the end of each PCR to check the specificity of the primers.

**Table 1:** Gene primer sequences.

Gene	Sequences	cDNA size	*T*_m_
NLRP3-F	*GGGCTTCTGCACCCGGACTG*	328	65°
NLRP3-R	*GGTGGTCCTGCTTCCACGCC*	328	65°
AIM2-F	*GTCACCAGTTCCTCAGTTGTG*	259	60°
AIM2-R	*TGTCTCCTTCCTCGCACTTT*	259	60°
NLRP1-F	*TCTCAGTGCCCAGGTGATTA*	151	60°
NLRP1-R	*TTGTCTCTGCTGCTTGAATGA*	151	60°
IPAF-F	*AAGGATGAAGGGCTGAAGGT*	159	60°
IPAF-R	*CGAAACTTGTAGGCTGACCA*	159	60°

### STATISTICAL ANALYSIS

Data were analyzed using a Mann–Whitney *U* non-parametric test or one-way ANOVA followed by a Dunnett’s Multiple Comparison or a Student’s *t*-test (SPSS program, version 17.0). Differences at a value of *p* < 0.05 were considered statistically significant

## RESULTS

### ETHANOL ACTIVATES THE NLRP3 INFLAMMASOME-COMPLEX FORMATION IN ASTROGLIAL CELLS FROM CEREBRAL CORTEX AND PRIMARY CULTURE

**FIGURE 2:**
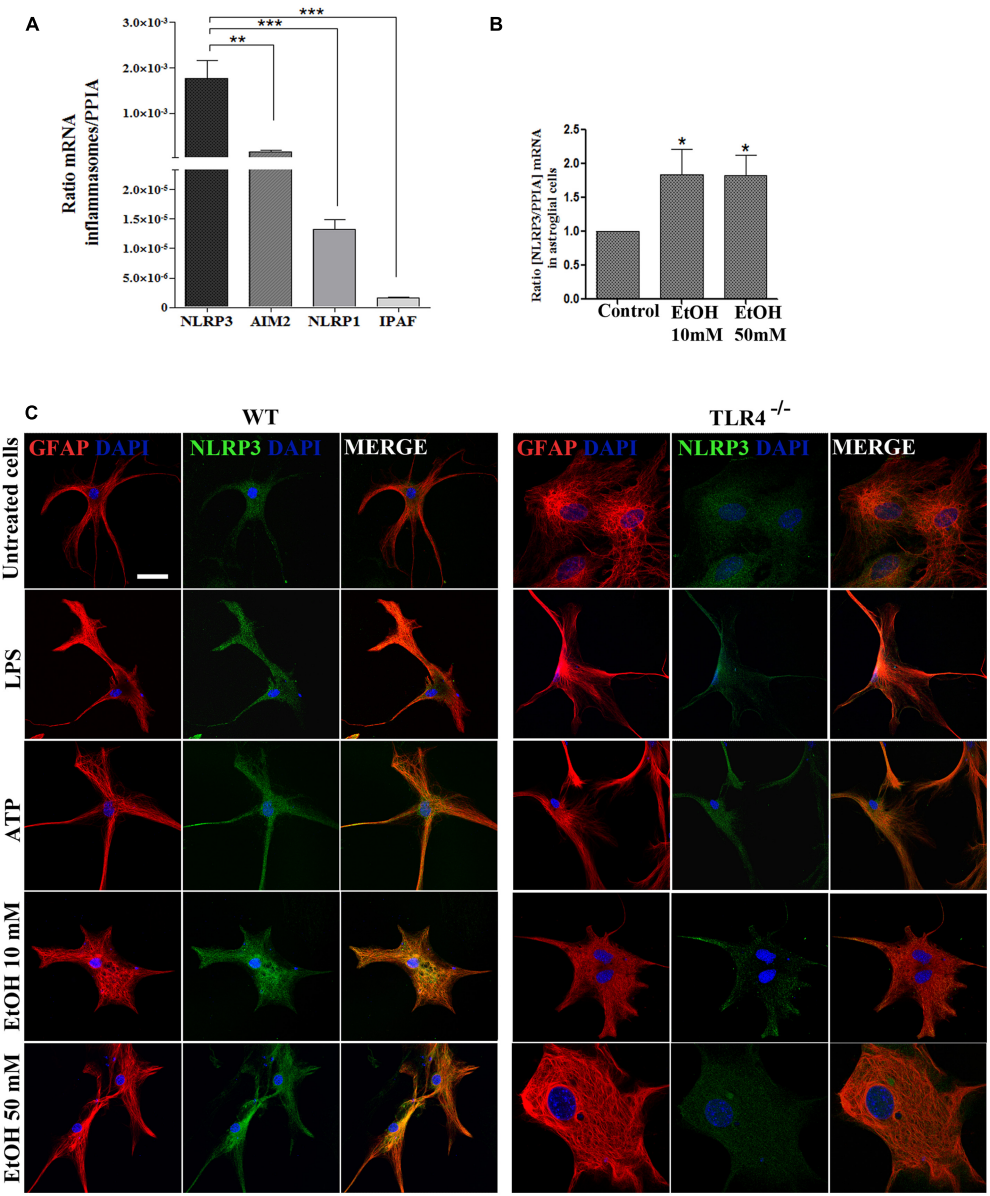
**Astrocytes in primary culture expresses high levels of NLRP3 mRNA.** **(A)** mRNA expression of NLRP3, AIM2, NLRP1, and IPAF inflammasomes in cultured astrocytes evaluated by RT-PCR. **(B)** Ethanol treatment (10 and 50 mM) for 24 h up-regulates the NLRP3 mRNA levels in the cultured astrocytes from TLR4 mice. We used *n* = 5–7 independent experiments and ratios were normalized with the PPIA housekeeping gene. Bars represent the [mean ±SEM]. *p < 0.05, **p < 0.01, ***p < 0.001 (Mann–Whitney *U* non-parametric test or a Student’s *t*-test). **(C)** Immuno-fluorescence of NLRP3 (green) and GFAP- (red) co-localization in the astrocytes from TLR4 and TLR4-knockout mice. Scale bar 20 μm.

Glial cells are important players in the immune response in the CNS (Farina et al., 2007). Our previous studies demonstrated that ethanol activates TLR4 signaling in astrocytes (Blanco et al., 2005; Alfonso-Loeches et al., 2010). We therefore wondered if ethanol was also capable of activating the inflammasome in astroglial cells to contribute to ethanol-induced neuroinflammation. To answer this question, we first evaluated whether ethanol treatment promotes caspase-1 activation and the release of interleukins IL-1β and IL-18 in both cerebral cortex (Ctx) area (**Figure 1E**) and in cortical astroglial cells (**Figure 2C**). The biochemical and immunohistochemical analyses revealed that chronic ethanol treatment increases the co-localization of caspase-1 with GFAP-positive cells (**Figures 1B,G**) and up-regulates the total caspase-1 activity levels (**Figure 1H**) in the cerebral Ctx of WT mice when compared with untreated animals (**Figures 1A,G**). We also determined interleukins IL-1β and IL-18 in the Ctx of the WT and TLR4-KO mice treated with or without ethanol for 5 months. **Figures 1I,J** shows that ethanol treatment up-regulates the levels of IL-1β and IL-18 in the Ctx of the WT mice. Conversely, the same ethanol treatment neither increased caspase-1 activation (**Figures 1C,D,G,H**) nor the levels of IL-1β and IL-18 (**Figures 1I,J**) in the Ctx of TLR4-KO mice.

To gain further insight into how ethanol affects inflammasome activation and which type of inflammasome was activated in astroglial cells, we used highly enriched astrocytes in primary culture, and the gene expression of different inflammasomes were assessed. An RT-PCR analysis revealed (**Figure 2A**) that although inflammasomes NLRP3, AIM2, NLRP1, and IPAF are expressed in astrocytes; the NLRP3 mRNA level was higher than the other inflammasomes, and the ethanol treatment (10 and 50 mM) was capable of up-regulating the NLRP3 mRNA levels (**Figure 2B**). The immunofluorescence studies also revealed that the NLRP3 protein expression is expressed in the cytosol of GFAP^+^ cells (**Figure 2C**), although higher NLRP3 expression are noted in WT astrocytes treated with LPS, ATP or ethanol (10, 50 mM).

**FIGURE 3:**
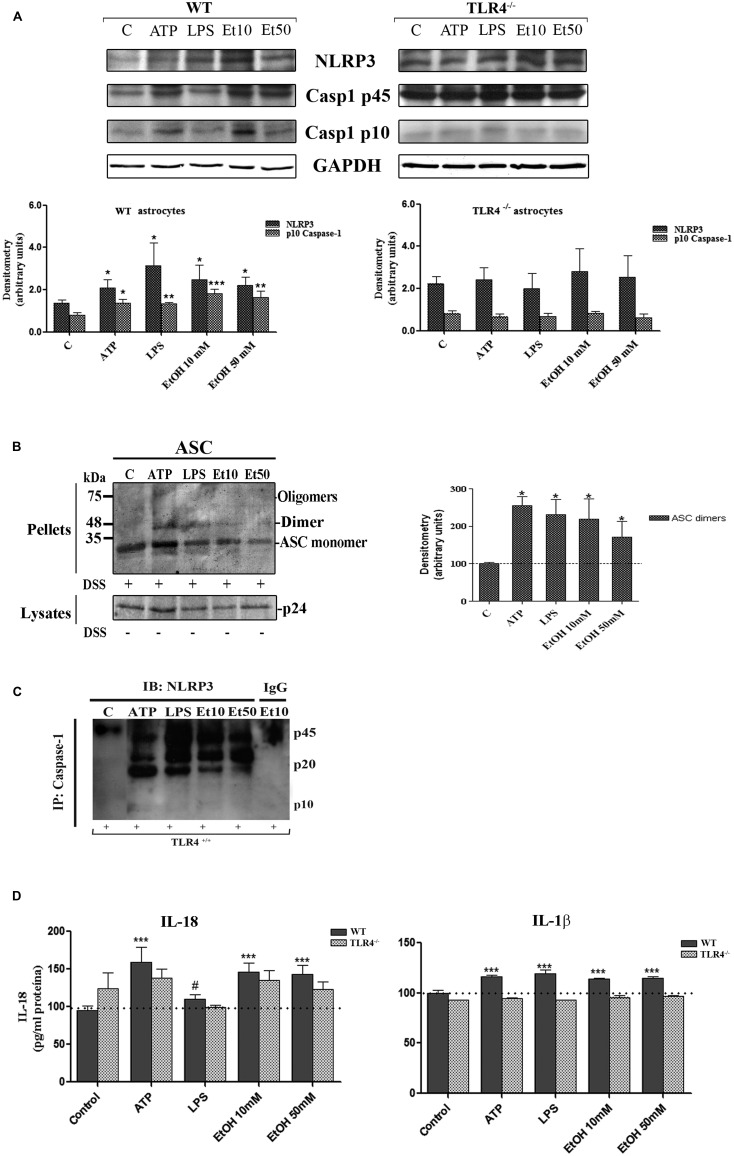
**Ethanol treatment activates the NLRP3 inflammasome complex in cultured astrocytes from TLR4-WT mice.** **(A)** Western blot analysis shows the NLRP3 and p10/caspase-1 active cleavage (the initiator caspase in pyroptosis) protein levels in the LPS-, ATP-, and ethanol- (10 and 50 mM) treated astrocytes. **(B)** We show ASC oligomerization and quantification of the ASC dimer by densitometry. Cells were lysed, pelleted by centrifugation and incubated with DSS for 30 min. The cross-linked pellets were resuspended in SDS sample buffer, and proteins were separated using 12% SDS-PAGE and Western blotted with anti-mouse ASC antibodies as described under Section “Material and Methods.” The presence of dimers and trimers was observed in the ATP-, LPS-, or ethanol-treated astrocytes correlating with a significative up-regulation of ASC dimers. **(C)** Cell lysates were collected and co-immunoprecipitated with the NLRP3 Ab (IP), and the immune complexes were detected by Western blot with Caspase-1 (IB). We show the presence of the p45 caspase-1 precursor and the active p20/p10 Caspase-1 in treated/untreated astrocytes. The mouse IgG was used as a negative control. **(D)** ELISA measured determined the IL-1β and IL-18 levels in the supernatant of the astrocytes treated with LPS, ATP and ethanol (10 and 50 mM) after 24 h. Non-significant differences were observed between the treated or non-treated TLR4-KO astrocytes. Values represent the mean ± SEM of 3–9 individual experiments. ^#^*p* < 0.06, *p < 0.05, **p < 0.01, ***p < 0.001 (Mann–Whitney *U* non-parametric test or a Student’s *t*-test).

The assembly of the NLRP3 inflammasome complex required adaptor protein ASC and its oligomerization to bring the receptor and zymogen pro-caspase-1 into close proximity, which led to caspase-1 activation, and to the cleavage of pro-IL-1β and pro-IL-18 into their active cytokine and pyroptotic cell death (Fernandes-Alnemri et al., 2007). Therefore, in order to evaluate if ethanol, in comparison with other inflammasome stimuli, was capable of inducing ASC oligomerization, the cleavage of caspase-1 and the production of IL-1β and IL-18, astrocytes were treated with LPS, ATP (a positive control of NLRP3 activation) and ethanol (10, 50 mM). **Figure 3** shows that ethanol and the LPS or ATP treatment promoted: (i) up-regulation of NLRP3; (ii) caspase-1 activation, as evidenced by the appearance of the 10 kDa active caspase-1-clevage peptide (**Figure 3A**); (iii) ASC oligomerization, as demonstrated by the notably presence of ASC dimers and some trimers (Fernandes-Alnemri and Alnemri, 2008; **Figure 3B**); (iv) the association between caspase-1 cleavage and NLRP3, as demonstrated by the Co-IP of both proteins with the appearance of the active p20/p10 caspase-1 in treated-astrocytes (**Figure 3C**); and (v) the production of pro-inflammatory cytokines IL-1β and IL-18 (**Figure 3D**). If we consider that ASC is an adaptor protein required for the activation of both NLRP3 inflammasome and caspase-1 (Fernandes-Alnemri et al., 2007), the results indicate that by inducing NLRP3 inflammasome complex activation and ASC-pyroptosome formation, ethanol was capable of inducing the innate immune response. Our results further suggest that activation of NLRP3/caspase-1 is associated with the TLR4 function since the astrocytes from TLR4-KO mice presented no response or a minimal one to different stimuli, including alcohol, on NLRP3 inflammasome activation ().

### ETHANOL-INDUCED MITOCHONDRIAL ROS FORMATION TRIGGERS NLRP3 INFLAMMASOME COMPLEX ACTIVATION

**FIGURE 4:**
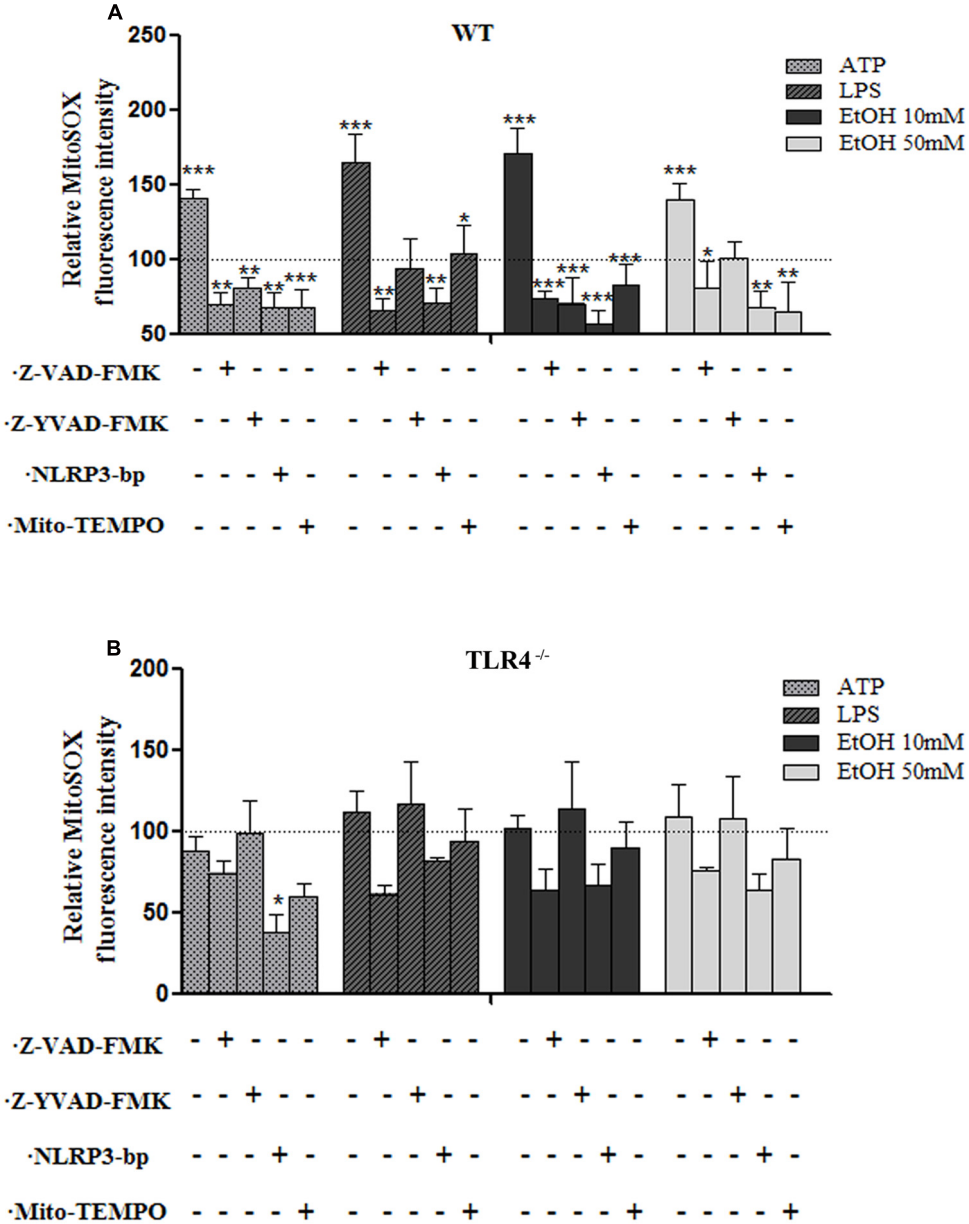
**The ethanol-induced mitochondria ROS production level measured by MitoSOX Red fluorescence intensity. (A)** A flow cytometry analysis shows that the ATP or LPS or ethanol (10 and 50 mM) treatments increased mROS generation in the WT astrocytes. Incubation with Z-YVAD-FMK, Z-VAD-FMK, the NLRP3 blocking peptide or Mito-TEMPO before and during treatments notably reduces mROS generation activation in WT astrocytes. **(B)** No significant changes in mROS generation were observed in the TLR4-KO-astrocytes incubated with the same inhibitors and treatments as used in the TLR4-astrocytes. Bars represent the (mean ± SEM) of 4–12 individual experiments. *p < 0.05, **p < 0.01, ***p < 0.001 (one-way ANOVA followed by a Dunnett’s Multiple Comparison Test or a Student’s *t*-test).

One of the mechanisms by which NLRP3 inflammasome can be activated is ROS generation (Tschopp and Schroder, 2010), and mROS formation seems particularly critical for NLRP3 activation (Zhou et al., 2011). We therefore explored the potential role of ethanol-induced mROS in NLRP3 inflammasome activation. To assess superoxide production by mitochondria in cultured astrocytes, we used the MitoSOX^TM^ Red reagent in live cells, a fluorogenic dye that specifically targets mitochondria in live cells, and a flow cytometry analysis. For these experiments, astrocytes were treated with ethanol (10 and 50 mM), LPS and ATP for 24 h in the presence or absence of Mito-TEMPO [a mitochondria (m) ROS scavenger] or a Z-YVAD-FMK (specific caspase-1 inhibitor) or z-VAD-FMK (inhibitor of caspase proteases) or a NLRP3 bp. Then mROS production was measured. The results indicate that treating WT astrocytes with ATP or LPS or ethanol (10 mM or 50 mM) markedly induced mROS production (**Figure 4A**). Notably, the mROS production induced by ethanol (10 and 50 mM) or LPS or ATP was significantly abrogated by Mito-TEMPO, z-YVAD-FMK, or Z-VAD-FMK by the presence of NLRP3 bp (**Figure 4A**). Strikingly, small, but non-significant, changes were noted in the TLR4-KO astrocytes incubated with ATP, or LPS or ethanol (**Figure 4B**). These results suggest that mROS generation induced by ATP or LPS or ethanol mediates NLRP3/caspase-1 inflammasome activation. Our findings also suggest crosstalk between TLR4 and NLRP3 inflammasome.

**FIGURE 5:**
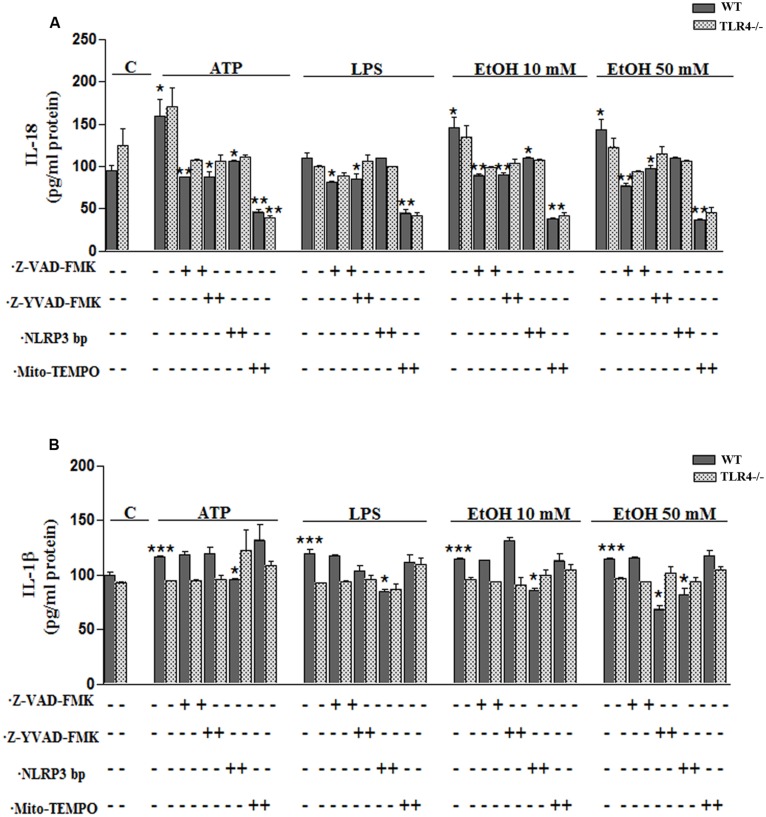
**Ethanol enhances the production of IL-18 and IL-1β in WT astroglial cells.** **(A,B)** Graphs represent the levels of IL-18 **(A)** and IL-1β **(B)** measured in the supernatant medium of the WT and TLR4-KO astrocytes treated with ATP, LPS or ethanol (10 and 50 mM) for 24 h. Incubation with Z-YVAD-FMK, Z-VAD-FMK, the NLRP3 blocking peptide or Mito-TEMPO, along with the different treatments, reduces, or even abolishes, the up-regulation of the cytokine release. No changes in the levels of cytokines were noted in the supernatant medium for the TLR4-KO-astrocytes for any of the treatments and inhibitors used, except for the Mito-TEMPO treatment at the IL-18 released levels. Bars represent the (mean ± SEM) expressed as a percentage (%) of 6–10 individual experiments. *p < 0.05, **p < 0.01, ***p < 0.001 (two-way ANOVA with *post hoc* Bonferroni’s correction).

To confirm the above results, we measured the levels of IL-1β and IL-18 in the medium of astrocytes incubated for 24 h with ATP or LPS or ethanol (10 and 50 mM), in the presence or absence of Mito-TEMPO or z-YVAD-FMK or Z-VAD-FMK or NLRP3 bp. According to the above results, ATP or LPS or ethanol (10 and 50 mM) treatment induces the production of IL-1β and IL-18 in the astrocytes supernatant, while the presence of Mito-TEMPO or z-YVAD-FMK or Z-VAD-FMK or NLRP3 bp abolishes most of the cytokine released in the cell medium (**Figures 5A,B**). However, the results presented in **Figure 5** illustrate that while both Z-YVAD-FMK and z-VAD-FMK were more efficient than NLRP3 bp or Mito-TEMPO in inhibiting IL-18 in all the treatments, the caspase-1 inhibitor did not completely abolish the release of IL-1β induced by ATP or LPS or ethanol, suggesting that this cytokine may be produced by other mechanisms (Hanamsagar et al., 2012). No significant variations in the levels of IL-1β or IL-18 were noted in the TLR4-KO astrocytes treated with ATP, LPS or even ethanol (**Figures 5A,B**).

### INFLAMMASOME STIMULATION INDUCES CASPASE-1 AND NLRP3 RECRUITMENT WITHIN THE MITOCHONDRIA IN ASTROCYTES

**FIGURE 6:**
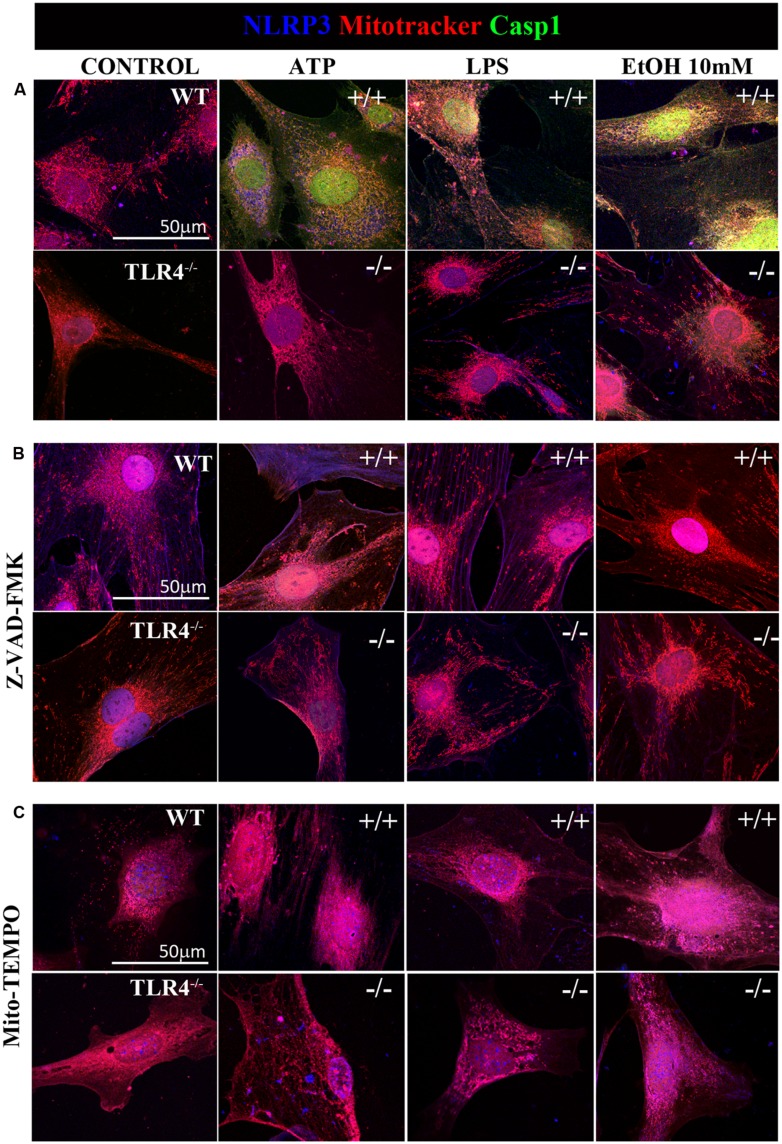
**Confocal images of NLRP3/caspase-1 co-localization within mitochondria of the astrocytes treated with ethanol, ATP, or LPS.** Microphotographs show that the ATP, LPS, or ethanol (10 mM) treatments promote the co-localization of NLRP3 inflammasome (blue) with active caspase-1 (green) within mitochondria (red) in the WT-astrocytes when compared with the untreated control astrocytes **(A)**. Astrocytes treated with Z-VAD-FMK **(B)** and with Mito-TEMPO **(C)** treatments do not induce caspase-1 activation in both WT and TLR4-KO astrocytes. We performed at least three independent experiments under each experimental condition. Representative pictures are presented.

Previous studies have shown that NLRP3 is located at the endoplasmic reticulum (ER) in the steady state and that it translocates to the mitochondria-associated ER membranes following stimulation (Zhou et al., 2011). Therefore, in order to gain further insights into ethanol-induced NLRP3 inflammasome activation, we used confocal microscopy to assess the location of NLRP3 under basal and stimulated conditions. We noted that under basal conditions, NLRP3 was located at the cytoplasm, but not within mitochondria (**Figure 6A**). We were unable to detect caspase-1 under the steady-state conditions. However, stimulation of astrocytes with ATP, LPS or ethanol (10 mM) led to the recruitment of caspase-1 and NLRP3 within mitochondria, as demonstrated by the overlap of caspase-1 and NLRP3 in the mitochondria (mitotracker staining). Strikingly, ATP and ethanol stimulation promoted higher caspase-1 levels within mitochondria than LPS. Inhibiting caspase-1 with Z-VAD-FMK (**Figure 6B**) or mROS with Mito-TEMPO (**Figure 6C**) mostly abolished NLRP3/caspase1 recruitment within mitochondria.

It is noteworthy that ATP, LPS, or ethanol (10 mM) did not promote NLRP3/caspase-1 activation and recruitment into the mitochondria in TLR4-KO astrocytes (**Figure 6**). Immunofluorescence studies with pro-caspase-1 and NLRP3 in untreated astrocytes and those treated with ATP, LPS and ethanol (10 mM) from WT and TLR4-KO mice also demonstrated that while stimulation with ATP, LPS or ethanol reduces the pro-caspase-1 levels and increases NLRP3 expression (**Figure 2**) and its co-localization in the WT cell cytoplasm, no changes in these parameters were observed in untreated or treated TLR4-KO astrocytes. In short, the results suggest that the by triggering pro-IL-1β production (Gross et al., 2011), TLR4 acts as a priming signal which, along with ROS production (second signal), promotes NLRP3 inflammasome activation. Lack of TLR4 signaling (TLR4-KO) was able to abolish the production of not only pro-IL-1, but also of other cytokines which might target to the mitochondria to initiate mROS production.

### ETHANOL-INDUCED NLRP3 INFLAMMASOME ACTIVATION TRIGGERS PYROPTOSIS AND APOPTOSIS

**FIGURE 7:**
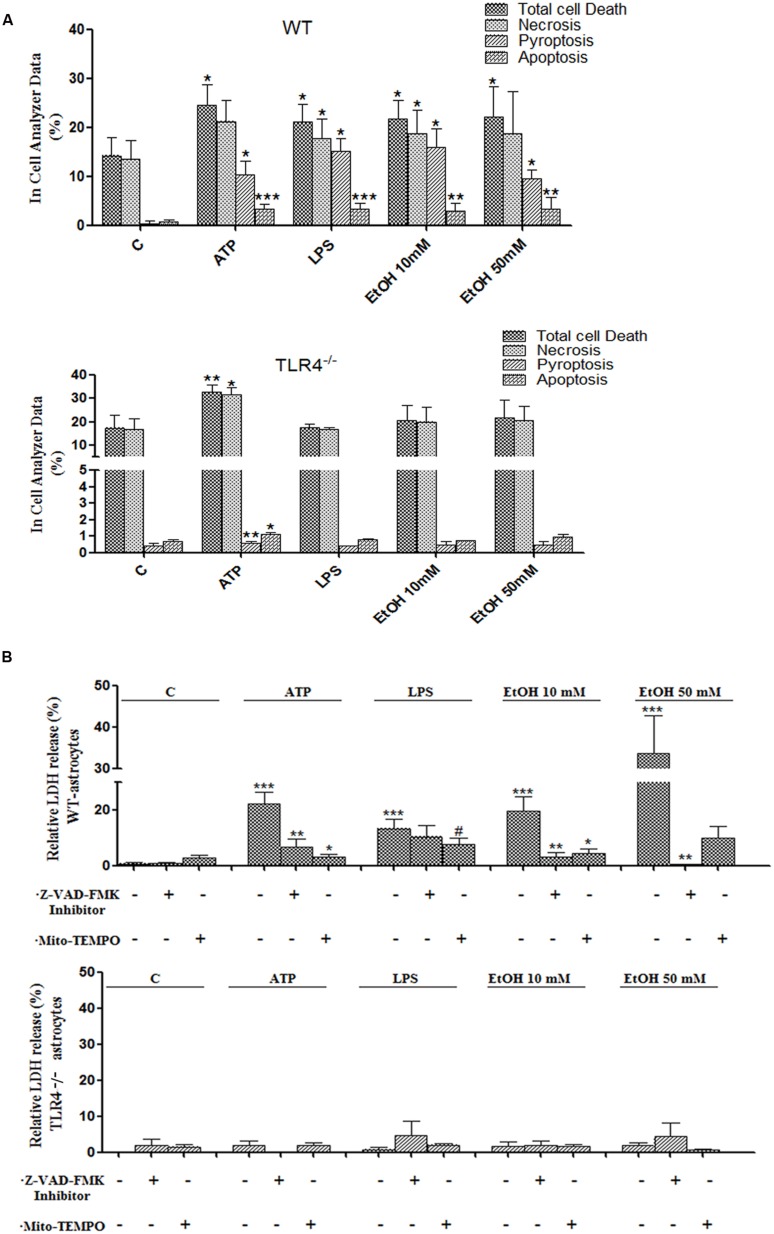
**Activation of the NLRP3 inflammasome complex triggers pyroptosis and apoptosis in ethanol-induced astroglial cells.** **(A)** Bars show the percentage of cell dead by pyroptosis, necrosis and apoptosis in astrocytes treated for 24 h with ATP, LPS, and ethanol (10 and 50 mM) evaluated by an In Cell Analyzer. Approximately 1000–5000 cells were analyzed/experimental condition. Bars represent the (mean ± SD). **(B)** LDH activity in the supernatant astrocytes was measured with different treatments. Bars represent the (mean ± SEM) of at least 6–10 individual experiments. *p < 0.05, **p < 0.01 (Mann–Whitney *U* non-parametric test).

Inflammasome-dependent caspase-1 activity has been shown to cause a rapid inflammatory form of cell death called pyroptosis, in which cytoplasmic content and pro-inflammatory cytokines, including IL-1β and IL-18, are released (Miao et al., 2011). However, apoptosis can also be induced by mitochondria stress and NLRP3 activation (Morizot, 2012), and our previous studies have indicated that ethanol can cause activation of caspase-3-dependent apoptosis and necrosis in the cerebral cortex (Alfonso-Loeches et al., 2010). Therefore, in order to evaluate whether ethanol-induced TLR4-dependent mitochondrial ROS production and NLRP3 activation promote pyroptosis and/or apoptosis, treated and untreated astrocytes were labeled with different staining and then, the proportion of those cells that died by apoptosis, necrosis or pyroptosis were evaluated using an In Cell Analyzer. As shown in **Figure 7A**, treatment of the astrocytes with ATP or LPS or ethanol 10 mM or ethanol 50 mM promotes 10.2, 15, 15.9, and 9.5% of pyroptosis (PI^+^ and FLICA 660-YVAD-FMK^+^ cells), respectively, when compared with untreated control cells.

Pyroptosis was also evaluated by measuring the levels of LDH release upon the different treatments. The results in **Figure 7B** illustrate that either ethanol or ATP treatments induces the release of cytoplasmic LDH in the cell medium. In all cases, pyroptosis was abrogated by Z-YVAD-FMK (a caspase-1 inhibitor) or Z-VAD-FMK or Mito-TEMPO treatments (**Figures 7A,B**). Notably, ATP, LPS or ethanol also induced a slight, yet significant increase in apoptosis (PI^-^ cells and increased staining with Hoechst 33342 due to DNA condensation). Thus, the percentage of apoptosis was 3.2, 3.3, 2.9, and 3.3% upon treatment with ATP, LPS or ethanol (10 and 50 mM), respectively (**Figure 7A**). No significant or minimal changes in pyroptosis or apoptosis were observed in treated TLR4-KO astrocytes.

**FIGURE 8:**
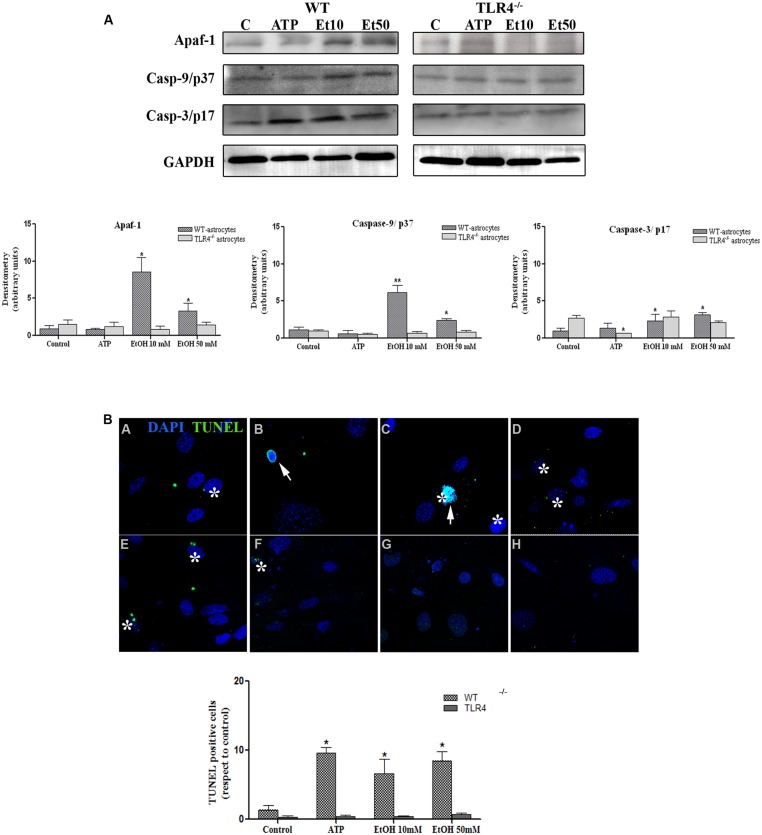
**Ethanol can also induce the apoptosome formation in astrocytes.** **(A)** The Western blot analyses of Apaf-1, the active fragments from caspase-9 (37 kDa) and caspase-3 (17 kDa), in the astrocytes treated with ATP (5 μM) or ethanol (10 and 50 mM). Bars represent the (mean ± SEM) of at least 6–10 individual experiments. *p < 0.05, **p < 0.01 (Mann–Whitney *U* non-parametric test or a Student’s *t*-test). **(B)** The percentage of apoptosis in the astrocytes incubated with ATP or ethanol (10 mM, 50 mM) for 24 h was assessed by a TUNEL assay in the WT and TLR4-KO astrocytes. Scale bar: 20 μm. Arrowheads show different apoptotic processes, blebbing and nuclei condensation. Asterisk show positive TUNEL cells. Bars represent the (mean ± SEM) of three individual experiments. *p < 0.05, **p < 0.01 (two-way ANOVA with *post hoc* Bonferroni’s correction).

Although apoptosis represents a small proportion of cell death induced by NLRP3 inflammasome activation, recent evidence has indicated that mitochondria stress can also lead to inflammation, NLRP3 activation and cell death by apoptosis (Morizot, 2012). We therefore evaluated the role of mitochondria ROS production and apoptosome formation by assessing the levels of Apaf-1 and active peptide caspase-9/caspase-3 in the astrocytes stimulated with ATP and ethanol. As shown in **Figure 8A**, both ATP and ethanol (10 mM and 50 mM) increase the levels of Apaf-1 and the active (cleaved) forms of caspase-9 and caspase-3 to trigger apoptosis, as confirmed by the TUNEL assay (**Figure 8B**). These results suggest that ethanol-induced NLRP3 inflammasome activation can trigger mainly pyroptosis, but may also induce apoptosis.

## DISCUSSION

Innate immune activation can occur in the nervous system in response to infections and/or tissue damage (Lampron et al., 2013). Two main receptors and signaling pathways are involved in this response, membrane receptors TLRs and cytoplasm sensors NLRPs, which regulate caspase-1 activity through inflammasome formation. Activation of TLRs has been shown to participate in brain infection and neurodegenerative disorders (Hanamsagar et al., 2012). However, less information is available on the role of inflammasome in brain damage and neuroinflammation, and the cellular source of inflammasome activation during brain infection or injury is lacking. In this study, we show that astrocytes express various inflammasomes, although NRLP3 expression is higher. We further demonstrate that ethanol as well as ATP or LPS, triggers the recruitment of NLRP3 and active caspase-1 within mitochondria by inducing mROS formation, which promotes the production of IL-1β and IL-18. The present findings also reveal crosstalk between TLR4 and the NLRP3 inflammasome complex since the elimination of the TLR4 function markedly reduces ethanol- or ATP-induced NLRP3 inflammasome activation and cytokines production.

NLRP3 is an intracellular protein complex composed of NLRP3, ASC, and pro-caspase-1, and it serves as a platform to activate pro-inflammatory cytokines IL-1β and IL-18 (Schroder et al., 2010). Recent structural studies have revealed the importance of ASC^PYD^/ASC^PYD^ and NLRP3^PYD^/ASC^PYD^ interactions during inflammasome activation (Lu et al., 2014). NLRP3 can be activated by pathogens and by a wide range of cytosolic DAMPs, including ATP, potassium eﬄux, alum, uric acid crystals and amyloid β (Jin and Flavell, 2010; Schroder et al., 2010) and is also involved in the pathogenesis of metabolic diseases, brain disorders, such as Alzheimer’s disease (Heneka et al., 2013), autoimmune encephalomyelitis (EAE), and it also contributes to ethanol-induced neuroinflammation in the cerebellum of ethanol-treated mice (Lippai et al., 2013). Although the cellular and molecular mechanisms of ethanol-induced inflammasome activation in the brain remain to be defined, we herein provide evidence that cultured astrocytes express NLRP3 and are capable of promoting ASC oligomerization to allow the recruitment of NLRP3 and caspase-1 activation (Lu et al., 2014) along with the production of IL-1β and IL-18 in response to ethanol, ATP or LPS. Inhibition of caspase-1 by Z-YVAD-FMK abolishes the production of IL-1β and IL18, suggesting the role of caspase-1 in NLRP3 activation and cytokine production.

Notably, while microglia appear to be the main player in NLRP3 inflammasome activation in the brain (Hanamsagar et al., 2012), recent studies have independently demonstrated that rat or human astrocytes express NLRP3 (Tezel et al., 2012) and NLRP2 (Minkiewicz et al., 2013) and are able to activate NLRP3 in response to DAMPs. Our *in vivo* immunohistochemical studies further support that *in vivo* ethanol treatment increases NLRP3 inflammasome activation in GFAP^+^ astroglial cells along with the production of IL-1β and IL-18 in WT mice cerebral cortex. According to these results, prior work from our laboratory has shown that ethanol promotes IL-1β production in both astrocytes (Blanco et al., 2005; Alfonso-Loeches et al., 2010) and microglia (Fernandez-Lizarbe et al., 2009) in culture, as well as in the cerebral cortex of ethanol-treated mice (Alfonso-Loeches et al., 2010).

Activation of the NLRP3 inflammasome by diverse stimuli usually requires two signals (Gross et al., 2011): the first signal, or priming signal is usually induced by TLRs/LPS signaling which triggers pro-IL-1β; and a second signal, induced by a diverse array of stimuli (e.g., ROS, ion membrane perturbations, ATP, etc.) and different mechanisms (Martinon, 2007; Petrilli et al., 2007; Qu et al., 2007). Recent evidence supported the critical role of mROS generation in NLRP3 activation (Nakahira et al., 2011; Shimada et al., 2012b). According to these findings, here we show that ethanol, similarly to other NLRP3 activators like ATP or LPS (Sutterwala et al., 2006), is capable of inducing mROS generation in astrocytes by triggering NLRP3 inflammasome activation, along with active caspase-1 maturation and the production of IL-1β and IL-18. The role of mROS in NLRP3 inflammasome activation has been supported by data demonstrating that blocking mROS or NLRP3, or the inhibition of caspase-1, abrogates both mROS generation and the up-regulation of IL-1β and IL-18 induced by ATP, LPS or ethanol. Indeed, previous studies demonstrate that ethanol-induced mROS generation participate in ethanol toxicity in both astrocytes (Gonzalez et al., 2007) and liver (Manzo-Avalos and Saavedra-Molina, 2010).

Cell death is ultimately caused by cellular innate immune response elicited by the inflammasome activation. We show that the activation of NLRP3 by either ATP, LPS or ethanol mainly triggers pyroptosis a caspase-1-dependent inflammatory form of programmed cells in which dying cells release their cytoplasmic content, including cytokines, into the extracellular space. Nevertheless, ethanol or ATP by inducing mitochondrial stress and mROS production, can also trigger apoptosis by activating Apaf-1, caspase-9, and caspase-3, and the apoptosome complex (Morizot, 2012). It is noteworthy that inflammasomes and apoptosomes are two mechanisms of the immune cells by which compromised cells are eliminated and share many similarities in their regulatory response to cellular stress (Latz et al., 2013). Yet whether these two mechanisms act independently or are linked, and whether their individual contributions depend on the intensity and type of cell stimuli (e.g., bacteria, virus, DAMPs; Doitsh et al., 2014), are uncertain (Latz et al., 2013). Nevertheless, *in vivo* evidence from the immunohistochemical data reported herein (**Figure 1**) and our previous studies support the *in vitro* finding since we observed that chronic ethanol treatment increases IL-1β levels and induces both apoptosis and caspase-3 activation, as well as necrosis, in the WT mice but not in the TLR4-KO mice cerebral cortex (Alfonso-Loeches et al., 2010).

Indeed, the present findings also support the role of TLR4 signaling in ethanol-induced NLRP3 activation, since the elimination of the TLR4 function in astrocytes abolishes ethanol- or ATP-induced NLRP3 activation. As commented above, a crosstalk between TLRs and inflammasome (Hanamsagar et al., 2012) has been reported. Activation of TLRs and the production of pro-IL-1β is the priming step for NLRP3 activation (Gross et al., 2011). Then, a second signal, induced by a diverse stimuli (e.g., ROS, ion membrane perturbations, ATP, etc.), promotes ASC-inflammasome oligomerization, caspase-1 activation, followed by the maturation and secretion of IL-1β and IL-18. A recent report has suggested that NLRP3 is activated by a two-step, deubiquitination mechanism initiated by TLRs signaling and mROS and that it is further potentiated by ATP, events which might explain how NLRP3 is activated by diverse danger signals (Juliana et al., 2012). Therefore, the lack of TLR4 function in TLR4-KO astrocytes not only eliminates the priming signal, as production of pro-IL-1β, but also the production of cytokines and free radicals-mediated by TLR4 signaling. These later events could, sensitize the mitochondria to induce ROS production and consequently NLRP3 activation and cell death by pyroptosis and apoptosis. Accordingly, recent evidence has demonstrated that TLR4 signaling induce mitochondria ROS production, contributing to the gastric cancer progression (Yuan et al., 2013). The functional role of TLR4 in the inflammasome activation is further supported by the demonstration that TLR4 deficiency not only protects ethanol-induced NLRP3 inflammasome activation and the induction of cytokines, but also attenuates the increased in ethanol-induced IL-1β production in the cerebellum (Lippai et al., 2013). We also noted that *in vivo* ethanol treatment abolishes the induction of IL-18 and IL-1β production in the cerebral cortex of TLR4-KO mice.

The role of TLR4 in ethanol-induced neuroinflammation and brain damage has been clearly demonstrated. Our previous studies have shown that by interacting with membrane microdomains “lipid rafts” in glial cells, ethanol can induce TLR4 dimerization and signaling to trigger the release of cytokines and inflammatory mediators (Blanco et al., 2005, 2008; Fernandez-Lizarbe et al., 2009). By using small interfering RNA (siRNA) or cells from TLR4-deficient mice (TLR4-KO), knockdown TLR4, abolish MAPK and NFk-B signaling pathways and the release of inflammatory mediators in glial cells (Fernandez-Lizarbe et al., 2009; Alfonso-Loeches et al., 2010). The *in vivo* relevance of these findings is evidenced by the demonstration that while chronic ethanol intake causes neuroinflammation, gliosis, demyelination and cell death (apoptosis and necrosis) in the cerebral cortex, TLR4-KO mice are protected against ethanol-induced brain inflammatory mediators, cell death and brain injury (Alfonso-Loeches et al., 2010, 2012). The present findings further support the role of TLR4 in ethanol-induced NLRP3 inflammasome activation and IL-1β and IL-18 production in glial cells, events that contribute to ethanol-induced neuroinflammation. To support our results, a recent study has demonstrated that ethanol-impaired neurogenesis is associated with induction of IL-1β and with inflammasome NALP1 and NALP3 activation in both neurons and astrocytes, and that these events can be blocked with both the IL-1β receptor and antagonist rIL-1Ra (Zou and Crews, 2012). Similarly, the intracranial administration of IL-1Ra prevents alcohol-induced inflammasome activation and the up-regulation of IL-1β and TNF-α in the cerebellum of ethanol-treated mice (Lippai et al., 2013). These results indicate the importance of ethanol-induced inflammasome activation and IL-1β production in the neuroinflammatory effects of ethanol.

## CONCLUSION

Taken together, our results demonstrate for the first time that, by promoting mROS generation, ethanol induces NLRP3/caspase-1 activation to trigger IL-1β/IL-18 production and cell death by pyroptosis and apoptosis, events that could contribute to neuroinflammation and brain damage induced by ethanol abuse. We further show crosstalk between NLRP3 and TLR4 since the elimination of TLR4 markedly diminishes ethanol actions on NLRP3 inflammasome activation and the production of the inflammatory cytokines IL-1β/IL-18. Our findings suggest that inflammasome activation may represent a new target in ethanol-induced neuroinflammation, and they support the potential role of IL-1Ra in the treatment of the neuropathological changes associated with alcohol abuse.

## AUTHOR CONTRIBUTIONS

Consuelo Guerri, Silvia Alfonso-Loeches, and Juan R. Ureña-Peralta conceived and designed the experiments. Silvia Alfonso-Loeches, Juan R. Ureña-Peralta, Maria José Morillo-Bargues, and Jorge Oliver-De La Cruz performed the experiments. Silvia Alfonso-Loeches and Juan R. Ureña-Peralta analyzed the data. Consuelo Guerri contributed reagents/material/ analysis tools. Consuelo Guerri, Silvia Alfonso-Loeches, and Juan R. Ureña-Peralta wrote the paper.

## Conflict of Interest Statement

The authors declare that the research was conducted in the absence of any commercial or financial relationships that could be construed as a potential conflict of interest.
